# Myeloid dermatosis with features of sweet syndrome and leukemia cutis: a case report

**DOI:** 10.1007/s00277-026-06949-7

**Published:** 2026-03-23

**Authors:** Evani Patel, Ajit Singh, Douglas Beach, Jennifer Morrissette, Gabriel Caponetti

**Affiliations:** 1 Department of Medicine, University of Pennsylvania, Pennsylvania Hospital, 800 Spruce St., Philadelphia, PA 19107 USA; 2 Department of Pathology and Laboratory Medicine, Hospital of the University of Pennsylvania, Philadelphia, PA USA

**Keywords:** Leukemia, AML, Sweet syndrome, Leukemia cutis, Mutation

## Abstract

Occult hematologic malignancies can initially present with cutaneous manifestations. We describe a 53-year-old woman with chronic hepatitis C and intravenous drug use presenting with a painful, progressive leg rash and worsening bicytopenias. A skin punch biopsy revealed neutrophilic dermatosis morphologically compatible with Sweet syndrome (SS), along with rare CD117+/CD34 − blastoid cells. Subsequent bone marrow studies revealed acute myeloid leukemia with mutated *NPM1*. This case highlights the diagnostic challenge of distinguishing SS from leukemia cutis (LC) when skin biopsies demonstrate mixed neutrophilic with rare blastoid cells. LC represents cutaneous infiltration of leukemic blasts, whereas SS represents a non-neoplastic, cytokine-driven process. Prompt immunophenotypic evaluation and genomic testing of myeloid dermatoses, particularly those with blastoid cells, can be critical in distinguishing SS from LC to provide significant insight on prognosis and therapeutic options. This case highlights the clinical and histopathologic spectrum of myeloid dermatoses and further emphasizes the relevance of skin biopsies in the diagnosis of cutaneous involvement by hematologic malignancies, such as myeloid neoplasms.

## Introduction

Cutaneous lesions occasionally represent the first sign of an occult hematological malignancy. Myeloid dermatoses mainly include leukemia cutis (LC) and Sweet Syndrome (SS). Both share some clinical and histopathologic similarities, making the diagnostic work-up challenging, especially in the setting of an unwell patient without a known pre-existing malignancy. Herein we present a case of a patient who presented with violaceous cutaneous plaques with skin biopsy findings consistent with SS, however also revealing rare blastoid cells. These skin findings were retrospectively considered to represent incipient involvement by LC after the patient was diagnosed with acute myeloid leukemia (AML) through a subsequent bone marrow biopsy.

## Case presentation

A 53-year-old female with a history of intravenous drug use (IVDU) and chronic hepatitis C (Hep-C) infection presented to the emergency department with a 2-day history of progressive right lower extremity swelling and lower calf pain that had not been preceded by trauma. An erythematous rash with ill-defined borders localized to the anterior and lateral sides of the right leg was also present.

**Fig. 1:**
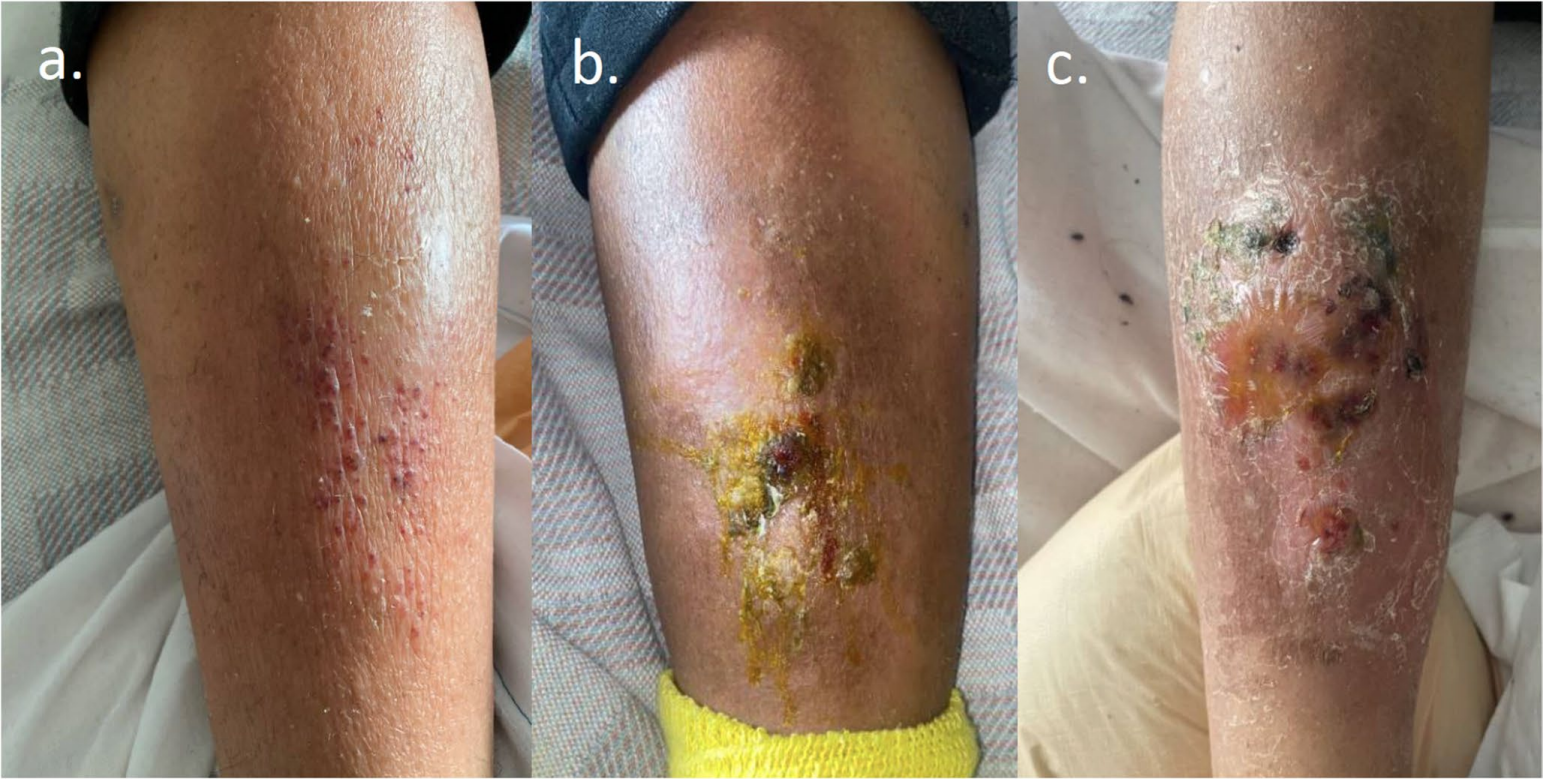
Rash progression throughout admission. **a** Right lower extremity (RLE) rash on day of presentation, **b** RLE rash on Day 16, **c** RLE rash on day 32

On admission, she was afebrile, normotensive, and had a normal heart rate. Laboratory studies revealed an elevated C-reactive protein (16.51 mg/L) and Erythrocyte Sedimentation Rate (100 mm/hr). A complete blood count on admission was remarkable for anemia (hemoglobin 7.6 g/dL) and a mildly low platelet count (150,000 K/uL). Although a computed tomography (CT) of the right leg showed extensive edema, an ultrasound of the same was negative for deep vein thrombosis. Given her history of IVDU, an echocardiogram was obtained and identified an aortic valve vegetation. Although blood cultures were negative throughout her admission, she was treated with vancomycin and ceftriaxone for culture-negative endocarditis, and a clindamycin course for suspected right leg cellulitis. During her admission, her platelets and hemoglobin down-trended, and antibiotics were changed to address the possibility of antibiotic-related cytopenias. No splenomegaly or evidence of hemolysis that could explain the cytopenias were identified, and Hep-C viral loads were stable. Within 30 days of admission her anemia and thrombocytopenia worsened, reaching nadirs of 20,000 K/uL platelets and a hemoglobin of 5.6 g/dL. Simultaneously, her skin rash progressed into an indurated plaque with clear borders (Fig. 1). A skin punch biopsy (PBx) and subsequent bone marrow (BM) studies were pursued.

**Fig. 2:**
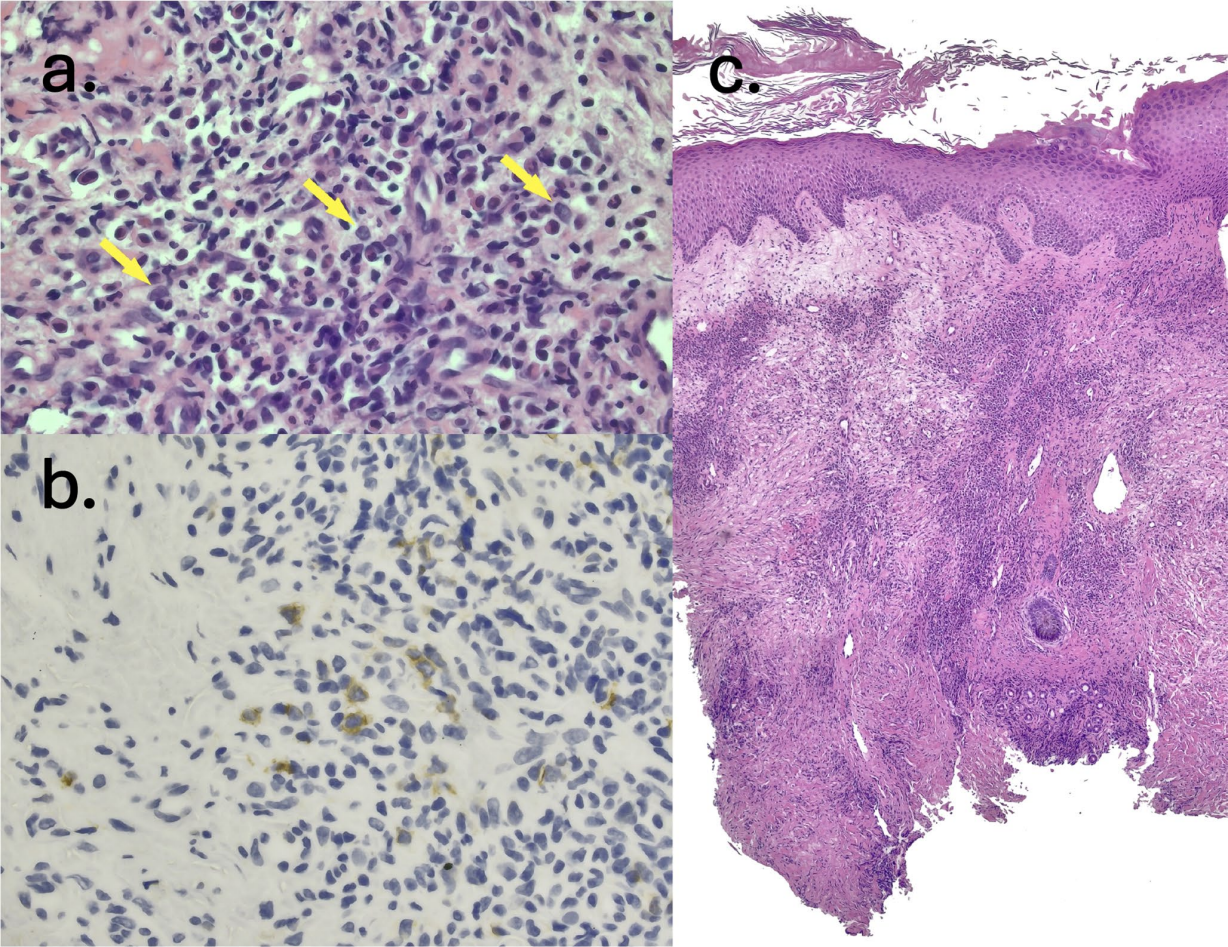
**a** H&E Skin Biopsy 40x, yellow arrows dictating rare blastoid cells in a neutrophil-rich dermal infiltrate, **b** CD117 (c-kit) immunohistochemistry stain, yellow-highlighted cells with CD117 + blastoid cells in a neutrophil-rich dermal infiltrate, **c** H&E Skin Biopsy 5x

PBx showed a perivascular infiltrate (Fig. 2c) of neutrophils and rare blastoid cells (Fig. 2a), without evidence of necrosis or vasculitis. Tissue cultures for microorganisms were negative. Immunohistochemistry (IHC) on rare blastoid cells identified expression of CD117 but not CD34 (Fig. 2b). A diagnosis of SS with rare blasts of uncertain significance was established a few days prior to receiving results of the initial BM studies.

Initial BM studies performed a few days following PBx demonstrated the presence of 10% CD117+/CD34- myeloblasts and 8% monoblasts. Cytogenetic studies revealed a normal female karyotype (46,XX). A gene sequencing panel of 308 genes in hematologic neoplasms showed disease-associated variants in *DNMT3A* (variant allele frequency, [VAF] 41%), *NPM1* (VAF 41%), *NRAS* (VAF 36%). Notably, there were no *UBA1* variants. An *IDH2* p.R140Q mutation was identified, however, present with a VAF below the limits of quantification. Thus, a diagnosis of AML with *NPM1* mutation was established.

Following PBx results, all antibiotics were discontinued, and oral prednisone was administered, which led to resolution of the leg rash. Given the results of the BM studies, the patient underwent two cycles of azacitadine-venetoclax and continued venetoclax on discharge. Subsequently, the patient was lost to follow-up until re-presenting 6 months later with worsening bicytopenia refractory to transfusions, and increased fatigue. Azacitadine-venetoclax was re-started with partial response, and she was then transitioned to decitabine. Repeat BM studies showed persistent AML with *NPM1* mutation. Her terminal hospitalization was complicated by sepsis and respiratory failure secondary to tracheal compression from a cervical mass which was biopsied as small cell carcinoma. She passed away two weeks later.

## Discussion

The diagnosis of SS involves the abrupt onset of painful papules and histologic evidence of neutrophilic infiltration without signs of vasculitis [1]. SS is hypothesized to be a reactive process involving cytokine signaling abnormalities due to myeloid dysregulation [2]. SS has been described in association with a variety of hematolymphoid malignancies, including AML, and VEXAS syndrome [3, 4]. In our case, due to the presence of a normal female karyotype, and the absence of characteristic cytoplasmic vacuoles in myeloid and erythroid precursors on bone marrow examination VEXAS was unlikely, and further testing for *UBA1* mutations reassuringly returned negative.

LC is distinct from SS as it involves skin infiltration of leukemic cells that are also found in the bone marrow and/or peripheral blood of patients with acute leukemia [5]. Retrospectively, the presence of rare blastoid cells within PBx of our patient represented a subtle involvement by AML and, therefore, may have been recharacterized as LC. Two important IHC markers in this case included CD34, a non-specific marker of all hematopoietic stem cells, and CD117, a protein oncogene that is most specific to primitive myeloid blast cells. CD117+/CD34 + blasts are commonly found in AML. However, a subset of AMLs can present with CD117+/CD34- blasts [6]. Although not pursued in our case, genomic evaluation of cutaneous infiltrates composed of blastoid cells can assist in establishing a diagnosis of LC by identifying disease-defining mutations such as *NPM1*. This is particularly helpful in cases where leukemic blastoid cells are CD34-negative, as commonly seen in *NPM1*-mutated AML, and when no prior diagnosis of acute leukemia has been established before skin biopsy [7].

Our case underscores the challenges encountered in clinical and laboratory practice when differentiating early leukemia cutis from reactive cutaneous infiltrates, including those associated with Sweet syndrome, especially in the setting of rare, CD34-negative blasts.

## Conclusion

SS exists on a spectrum of myeloid dermatoses, with LC being perhaps its most aggressive mimicker. Distinction between these two entities can be challenging at the clinical and histopathologic level. Multi-disciplinary management, including the collection of punch biopsies, evaluation by immunohistochemistry, and in some cases, genomic testing should not be delayed if rashes persist. Finally, clinical suspicion of Sweet syndrome should prompt consideration of leukemia cutis in the differential diagnosis.

## Data Availability

The data that support the findings of this study are available from the corresponding author upon reasonable request.
